# Dutch dismissal practices: characteristics, consequences, and contrasts in residents’ case law in community-based practice versus hospital-based specialties

**DOI:** 10.1186/s12909-024-05106-w

**Published:** 2024-02-19

**Authors:** Judith Godschalx-Dekker, Walther van Mook

**Affiliations:** 1 Department of Psychiatry and Medical Psychology, GGZ Central, Flevoziekenhuis, Almere, The Netherlands; 2 Department of Intensive Care Medicine, Academy for Postgraduate Training, Maastricht UMC+, and School of Health Professions Education, Maastricht University, Maastricht, The Netherlands

**Keywords:** Aptitude, Assessment, CanMEDS, Dismissal, Procedures, Remediation, Safety

## Abstract

**Background:**

In the Netherlands, 2 to 10% of the residents terminate training prematurely. Infrequently, termination of training is by dismissal. Incidentally, residents may disagree, dispute and challenge these decisions from the programme directors. Resident dismissal is always a difficult decision, most commonly made after, repeated assessments, and triangulation of the resulting assessment data and one or more remediation attempts. Nevertheless, the underlying reasons for dismissal and the policies for remediation and dismissal may differ between training programmes. Such differences may however impact the chance of remediation success, the chance of dismissal and subsequent residents’ appeals.

**Method:**

We included a total of 70 residents from two groups (community-based and hospital-based specialties) during 10 years of appeals. Subsequently, we compared these groups on factors potentially associated with the outcome of the conciliation board decision regarding the residents’ dismissal. We focused herein on remediation strategies applied, and reasons reported to dismiss residents.

**Results:**

In both groups, the most alleged reason to dismiss residents was lack of trainability, > 97%. This was related to deficiencies in professionalism in community-based practice and medical expertise in hospital-based specialties respectively. A reason less frequently mentioned was endangerment of patient care, < 26%. However, none of these residents *accused* of endangerment, *actually* jeopardized the patients’ health, probably due to the vigilance of their supervisors. Remediation strategies varied between the two groups, whereas hospital-based specialties preferred formal remediation plans in contrast to community-based practice. A multitude of remediation strategies per competency (medical expertise, professionalism, communication, management) were applied and described in these law cases.

**Discussion:**

Residents’ appeals in community-based practice were significantly less likely to succeed compared to hospital-based specialties. Hypothesised explanatory factors underlying these differences include community-based practices’ more prominent attention to the longitudinal assessment of professionalism, the presence of regular quarterly progress meetings, precise documentation of deficiencies, and discretion over the timing of dismissal in contrast to dismissal in the hospital-based specialties which is only formally possible during scheduled formal summative assessment meetings.

**Supplementary Information:**

The online version contains supplementary material available at 10.1186/s12909-024-05106-w.

## Background

Insufficiencies in residents are inherently tied to postgraduate medical education. Residents are in training to acquire knowledge and skills when practicing patient care, hopefully developing into excellent medical professionals with regard to independent and collaborative practice within their specialisation. However, a subsequent number of residents struggle. This struggle is at least temporary in approximately 4–7% of the residents in internal medicine [1, 2, 3] 18–21% of the residents in surgery and related specialties [4–9], and in 9–23% of the residents in general practice [10–12]. In general, residents struggle most with acquiring competencies in medical expertise, communication, and professionalism [13–16]. 

Presumably, at least some of these residents’ supervisors and programme directors may struggle as well, for example to recognize or acknowledge the resident’s problematic performance, to provide appropriate guidance and remediation [17], and, as an ultimum refugium, to restrain the practice of residents unapt for their specialty if improvement does not occur. This struggle is referred to as the “failure to fail” problem [18], mainly described related to regular assessment moments by supervisors of medical school undergraduates [19, 20]. Reluctance to address poor resident performance and to act on demerit could ultimately lead to “failing to fail” residents subsequently compromising health care quality [21]. Failure to fail also applies to the inability of medical educators to restrain training progress and graduation in students and residents who are considered unapt or perform poorly [22]. Assumably, this inability is highly related to unavailability of documentation about previous performance, remediation provided, associated administrative procedures and/or perceived legal barriers [18, 23, 24]. 

In the Netherlands, implementing competency-based education increased the assessment of residents’ aptitude during the past ten years. If a resident’s aptitude is in doubt, the resident is encouraged to remediate. Formal remediation is offered to the poorly performing resident who persistently fails to meet the criteria of one or more CanMEDS competency domains. If despite resident remediation, inadequate aptitude persists, the resident could ultimately be dismissed by the programme director [25–27]. Eventually, programme directors dismiss only a small percentage of residents, between 0.1% and 2.6% of the enrolled residents, with a mean percentage of 0.6% [2, 13, 16]. Factors associated with dismissal of residents have not yet been fully elucidated.

The reasons underlying dismissals of residents are thus an unexplored area in medical education research, however relevant for programme directors and educators to optimize recognition of residents’ performance problems as well as to offer appropriate remediation. In principle, dismissal is confidential for the persons involved, making it impossible to learn from these situations for medical education in general. Medical education may profit from the wisdom of programme directors who faced the problem of failure to fail and nevertheless decided to dismiss a resident who disputed their decision. Fortunately, learning from publicly available cases challenging dismissal is possible, though such cases have so far not systematically been analysed and described before. Exceptionally, Dutch decisions about residents who challenge their dismissal can be subject of such research, because the case law database from 2011 till 2020 is publicly accessible.

No previous research exists on the outcome of these dismissal procedures, especially related to the types of remediation attempted, or the differences in dismissal policy applied in residency training for different types of specialties. Differences may nevertheless exist between specialties in this regard. For example, Dutch General Practitioner (GP) training has a long history of longitudinal assessment of aptitude [10], whereas most other postgraduate training programmes do not. Other characteristics of educational and dismissal policy in community-based practice, such as general practice may differ between hospital-based specialties as well. In addition, no research exists on residents’ argumentation to avoid dismissal in general, and for the two practices specifically. Therefore, we studied the publicly available appeals of Dutch dismissed residents to determine factors potentially influencing their outcomes. Our research questions were which remediation strategies were described, what reasons were denominated to dismiss residents from the training programme, and was there a difference between community-, and hospital-based specialties in dismissal rates and remediation strategies?

## Methods

We performed an open-source retrospective study of cases of dismissed residents who appealed the programme director’s decision to dismiss. We studied both quantitative and qualitative characteristics of these law cases in two different types of residency training programmes, community and hospital-based specialties.

### Context and setting

In the Netherlands, the postgraduate training programme director is responsible for the assessment of the residents’ performance [28]. If poor performance persists or causes critical incidents threatening patients’ safety or physician moral standards, the resident could ultimately be dismissed from the programme. The dismissal procedure for residents in training in community-based practice differs from hospital-based specialties (Table 1).

**Table 1:** Differences in educational law procedures

Training characteristics	Community Practice	Hospital Specialties
Nominal years of training	3 years	4.5–6 years
Programme	2 years of supervision with a GP and 1 year of rotations in between in paediatrics, emergency medicine and psychiatry	Rotations of 6 to 12 months
Nationwide knowledge test	Yes	No
Longitudinal mentorship	Yes	Optional
Reflection classes	Yes	No
Conditional passing	Optional	No
Supervisor disconnection	Optional in GP	Exceptional
Dismissal procedure		
Actor of dismissal	Head of residency training	Programme Director
Timing of dismissal	Flexible at any time in residency because of conditional passing or compelling reasons (such as untrainability)	Fixed moment determined to assess aptitude, unless critical safety incident or other compelling reason
Mediation initiated by	Institution with external mediator	Centralized Educators Committee

Legend: this table shows noticeable differences of design and regulation of training programmes for residents in Community Practice (general practitioner and nursing home doctor residents) and Hospital-based Specialties [10, 12, 29–31]

Reflection classes: classes designed to teach the residents to reflect on difficulties experienced during independent practice. Conditional passing: the training programme lets the resident in training pass to the next year of training, however, this allowance is conditional. The resident needs to fulfil certain conditions within a limited period of time, mostly within 3 to 6 months, or else the training program will be terminated. Supervisor disconnection: both the supervisor and the supervisee are entitled to disconnect their master-apprentice relationship prematurely. In such case the supervisee is entitled to continue the training at another general practitioners office with a new supervisor

In community-based practice, the head of residency training is mandated to dismiss the resident; in hospital-based specialties, dismissal is mandated to the programme director. The resident who challenges a dismissal decision, first requests mediation. In community-based practice, a request that a priori is judged feasible to all stakeholders is passed on to an external mediator. In hospital-based specialties, conflict resolution is a task of the centralized educators’ committee. This centralized educators’ committee is an advisory and supervisory body of programme directors. The committee examines whether it is possible to settle the dispute. Unless a settlement is reached, both groups of residents, in community-based practice and hospital-based specialties alike, may request conciliation from the board of The Royal Dutch Medical Association.

### Conciliation board

The conciliation board of the Royal Dutch Medical Association is a national conciliation board, with two legal professionals (chairman and clerk), a programme director, and a resident, both preferably from the same specialty, but both from another institution. The conciliation board may decide upon a resident’s request. In case a resident appeals dismissal, the board does not judge the aptitude for residency but considers whether the programme director or the head of residency training, made a deliberate and careful decision. The conciliation board’s decision is binding for both parties, but the resident is entitled to pursue further legal action before the court of first instance. However, residents seldomly do so and the court limits itself to a procedural review [32]. In response to a resident’s request, the conciliation board may decide that the resident is entitled to continue residency training, for example with more intensive supervision and additional (workplace based) evaluations, occasionally in another training institution. The conciliation board case collection is the only accessible nationwide collection of cases of dismissed residents, about whom the reasons for dismissal are carefully and structurally described. Each annual report contains anonymized cases decided by the board that year and made publicly available online in Dutch by the conciliation board itself.

### Data collection

In February 2021 we perused the website and annual reports from 2011 to 2020 of the conciliation board of The Royal Dutch Medical Association. We selected all law cases of residents from community-based practice (general practice and nursing home doctors, who especially treat elderly patients outside of the hospital, have similar dismissal policies and design of training programme) and selected a matched number of law cases of residents from hospital-based specialties, by systematic and randomized exclusion of available law cases.

We performed quantitative and qualitative analysis of the law cases based on the following research questions. Our research questions for the quantitative analysis were: were there significant differences between the success rates of residents’ appeals at the conciliation board about dismissal in community-based practices and hospital-based specialties, and if so, which characteristics might be associated with these differences? Our research questions for qualitative analysis were: which remediation methods were described, and what reasons were denominated to dismiss residents from training in both community-based practice and hospital-based specialties?

### Analysis

We quantitatively and qualitatively compared the two groups of Dutch dismissed residents regarding the residents’ characteristics and programme policy characteristics, as well as the success rates of a dismissal appeals. Residents’ characteristics such as gender, number of deficient CanMEDS, the reasons for dismissal, and years in training at dismissal, were supplemented with programme policy characteristics such as the number of dismissals respected by the conciliation board, the types and frequencies of remediation and mediation previously tried, technicalities and violated educational law procedures.

#### Quantitative analysis

Descriptive statistics were performed using Excel version 2202 and included the mean number and percentages of deficient CanMEDS competency domains, and mean years of training at dismissal. To examine possible gender effects [33], using a X^2^-test, we then compared the percentages of men on average in training from 2010 to 2018 between specialties of dismissed residents (alpha < 0.01) [32, 34]. We used Jamovi 2.2.5 for inferential statistics. We performed X^2^-test or Fisher-exact tests depending on *n* < 5, *p* < 0.05 to determine whether case law characteristics differed between community-based practice and hospital-based specialties. We performed an independent sample t-test (t < 0.05) to determine whether the duration of training or the mean number of deficient CanMEDS differed between each group.

#### Qualitative analysis

The principle investigator performed a systematic within-case analysis of the law cases. She transcribed verbatim the facts of the residents’ education, position statements of the resident and the programme director and the considerations of the conciliation board, that were related to the research question. She arranged described remediation methods applied to different CanMEDS competency domains. She collected the arguments the programme directors used for a dismissal, such as compelling reasons and untrainability and she determined whether and why these reasons were respected by the conciliation board.

## Results

A total of 35 resident appeals were selected in both community-based and hospital-based specialties. Dismissed residents from community-based practice were more frequently male when compared to all residents in training for community-based practice (Table 2). Dismissed residents from hospital-based specialties were not different concerning gender when compared to all the residents in training for hospital-based specialties.

**Table 2:** Community-based Practice versus Hospital-based Specialties characterized by number and gender of residents

	Residents in case law			Case law / Residents in programmes		Residents in training programme		
	N	N male	% male	%	Gender X^2^	N	N male	% male
Community Practice	35	21	60.0%	1.5%	0.002	2274	532	23.4%
General Practitioner	24	12	50.0%	1.2%		1978	471	23.8%
Nursing Home Doctor	11	9	81.8%	3.7%		296	61	20.6%
Hospital Specialty	35	13	37.1%	1.0%	0.626	3409	1458	42.8%
Internal Medicine	5	1	20.0%	0.3%		1913	790	41.3%
Radiotherapy	4	0	0%	4.1%		97	25	25.8%
Surgery	3	1	33.3%	0.8%		398	226	57.1%
Dermatology	5	0	0%	3.3%		151	38	25.2%
Anaesthesiology	7	5	71.4%	1.5%		466	179	38.4%
Radiology	11	6	54.5%	2.9%		384	199	51.8%
Total	70	34	48.6%	1.2%		5683	1990	35.0%

Legend: This table shows the characteristics of case law decided by the conciliation board from 2011 to 2020 in the Netherlands compared to mean number of residents in training from information published by the capacity body

Community Practice: n residents in training weighted mean from 2010 to 2018

Hospital Specialty: n residents in training weighted mean from 2010 to 2019

### Comparison of resident cases from community-based and hospital-based specialties

The conciliation board confirmed the dismissal decisions in community-based practice significantly more frequently (X^2^, df1, *p* = 0.034) when compared to hospital-based specialties (Table 3). Cases of residents in community-based practice differed in characteristics from those in hospital-based specialties. The number of residents addressed as unprofessional, and the preferential use of other forms of remediation, over formal remediation plans, was higher in community-based practice. Furthermore, cases from community-based practice were often more accurately documented regarding the severity of residents’ deficiencies. Finally, community-based specialties used a mediator in the disputes more often and received fewer residents’ complaints about the educational climate during training.

**Table 3:** Characteristics potentially relevant to the programme director’s decision to dismiss a resident from training

	Community	Hospital	*p < 0.05 (test)
Confirmation of dismissal decision by conciliation board	29 (83%)	21 (60%)	0.034* (X^2^)
Gender = Male	21 (60%)	13 (37%)	0.056 (X^2^)
Years of training (mean) at dismissal	2.01	2.17	0.594 (t-test)
No deficient CanMEDS (mean)	3.4	3.1	0.289 (t-test)
Professional	31 (89%)	18 (51%)	0.001* (Fisher)
Communicator	27 (77%)	22 (63%)	0.192 (X^2^)
Manager	18 (51%)	22 (63%)	0.334 (X^2^)
Medical Expert	23 (66%)	28 (80%)	0.179 (X^2^)
Remediation plan			
Formal	14 (40%)	21 (60%)	0.094 (X^2^)
Other forms	20 (57%)	10 (29%)	0.016* (X^2^)
Reasons to dismiss the resident			
Untrainable	34 (97%)	35 (100%)	0.314 (X^2^)
Compelling reasons	9 (26%)	6 (17%)	0.382 (X^2^)
Mediation			
Unfeasible	6 (17%)	16 (46%)	0.010* (X^2^)
Mediator	29 (83%)	2 (6%)	< 0.01* (Fisher)
Second opinion	1 (3%)	4 (11%)	0.356 (Fisher)
Other characteristics, technicalities and violated procedures			
Premature dismissal	11 (31%)	10 (29%)	0.794 (X^2^)
Lacking documentation about severity of deficiencies	1 (3%)	8 (23%)	0.028* (Fisher)
Unclarity of the remediation plan	1 (3%)	3 (9%)	0.614 (Fisher)
Lack of residents’ guidance	3 (9%)	9 (26%)	0.062 (Fisher)
Resident temporarily on sick leave	14 (40%)	13 (37%)	0.806 (X^2^)
Claiming an unsafe educational climate	1 (3%)	9 (26%)	0.013* (Fisher)
Dissenting opinions in the training staff concerning dismissal	4 (12%)	2 (6%)	0.673 (Fisher)

Legend: This table shows a comparison of the frequencies of characteristics of law cases of dismissed residents from community-based practice and hospital-bases specialties. Frequencies were tested with X^2^ or Fisher-exact or student test, depending on the frequencies < 5 and type of variables (binary or continuous). A formal remediation plan was a timed intervention of 3 to 6 months as specified in the Dutch residency training rules. We distinguished several technicalities evidenced by the conciliation board in their considerations or dictum, such as when a program director decided to dismiss a resident at a premature time, lacked sufficient and coherent documentation about feedback given to the resident about the severity of deficiencies, lacked a clear plan or outline for a remediation approach (without specific goals, conditions, methods or tools about what the resident should improve), lacked clear conditions or actual guidance for the resident. Moreover, an unsafe educational climate means a claim made by the resident that the working- or learning climate at the institution or training program was considered psychologically unsafe. Such a claim could be agreed on by the program director and/or evidenced by the conciliation board in their considerations or dictum

#### Remediation strategies

The remediation strategies per deficiency in CanMEDs competency differed per CanMEDs competency in the overall cohort of law cases (Table 4). Remediation regarding professionalism included reflection-promoting activities, such as reflective writing and coaching. Remediation regarding communication addressed three specific problems. These included problematic interaction (for example with video supervision or roleplay), problems regarding comprehending or expressing Dutch language (for example with courses and tests to identify and exercise specific language difficulties), or the lack of quality or quantity of writing coherent progress letters about patients (for example with letters’ correction or sample checks). Remediation regarding management included minimizing complex multitasking, and more gradually increasing the demands of the work environment. Community-based practice uses remediation strategies such as: assessment resits, changing the supervisor of the resident, extending the duration of the residency training, and allow provisionally and conditionally passing the residents’ to the next programme year, risking termination of the training if the resident was unable to achieve goals specified. These conditions for passing were most often measurable timely targets. Hospital-based specialties more often resorted to a formal remediation plan, as specified by the Dutch residency training rules of minimum 3, but mostly 6 months.

**Table 4:** Remediation strategies per CanMEDs competency

CanMEDS competency	Remediation strategies
Professional	Mentor, (role-modelling) tutor, coach, team coach, intervision
	Additional talks with programme director or training staff
	Writing reflection assignments
	Career advice or career coaching
	Strengths and weakness analysis, psychological assessment
	Psychiatrist, psychotherapist or psychologist
Communicator	Interaction: video assessments, roleplay, communication training.
	Language: logopaedic, language course.
	Written: letter correction.
Manager	Reducing multitasking such as a training programme break, quitting research projects, adapted outpatient clinics, rotations in a smaller hospital, working parttime, not participating in irregular shifts.
Medical Expert	Extension of training duration, passing with specified conditions, such as setting measurable timely targets.
	Competency matrix or personal performance development plan.
	Clinical practice assessments, help of an educational expert.
	Exposure to activities that currently lack quality or experience.
	Resit of assessments, exams or rotations.
	Checks, direct strict supervision.

Legend: This table shows the remediation strategies for several, most often deficient CanMEDS, per CanMED in residents ultimately dismissed from the programme

#### Reasons to dismiss residents

In general, the most alleged reason to dismiss a resident found in our case law collection (Table 3), was lack of trainability (97-100%). This was related to deficiencies in professionalism in community-based practice and medical expertise in hospital-based specialties respectively. Compelling reasons such as co-occurring endangerment of patient care were far less common (17–26%). Such compelling reasons were identified during situations in evening or night shifts, and/or emergency or intensive care medicine (Table 5). Concerns for patients’ safety were expressed in at least eight cases. Presumably, these patients did not actually suffer from the reported incidents, because the training staff discovered these incidences in time, and was able to prevent potential harm. The conciliation board respected compelling reasons to dismiss in most (12 of 15) cases but refused this reason whenever the threat to patient safety was not substantiated enough. In those cases, the decision to dismiss was delayed and/or disproportional, so that the dismissal did not immediately follow after the safety incident, or ‘unsafety’ was an undifferentiated feeling of the hospital staff. The board, however, did respect other reasons to dismiss, for example when nursing and training staff no longer supported the residents’ participation in the workforce, residents’ unreliability, or residents’ sick leave. Sick leave was considered to prevent adequate rehabilitation and remediation. Sick leave was far more common in the case law herein studied (37–40% versus 14%, X^2^, df1, *p* = 0.001) when compared to Vermeulen (et al., 2016) [10] who studied a whole community practice resident cohort in a single Dutch institution.

**Table 5:** Compelling reasons to dismiss

Case number	Reason explained by programme director or head of residency training	Respected?
	Patient safety	
2013-63209 C	Incident during a weekend shift, concerning patient safety.	Yes
2019-6 C	Three types of incidents such as multiple faults in prescribing medication, not taking concerns of nurses seriously concerning wound care, forgot to attend to a family conversation.	Yes
2017-63269 C 2017-63284 C	Insufficient assessment of the seriousness of patient problems (knowledge from the books not being able to applicate to clinical reasoning and decision making in practice).	Yes Yes
2017-63281 H	Two incidents risking patient safety on the ICU.	No
2016-63263 H	Complaints and missed diagnosis, behaviour during shifts.	No
2019-4 H	A pattern of dysfunctioning discarding continuity and safety of patient care.	No
2013-63211 H	Carelessness with radiation therapy leading to concerns about patient safety.	Yes
	Unreliability	
2016-63264 C	Forgery about attending a conference.	Yes
2012-63192 C	No show on an emergency services shift, on top of other professionalism lapses.	Yes
2013-63198 C	Resumé fraud.	Yes
	Losing support from colleagues or staff	
2013-63203 C	Level of performance and lack of ability to work independently at the emergency medicine services.	Yes
2013-63212 C	Incidents resulting in interruption of the rotation in emergency medicine.	Yes
2016-63255 H	Severity of deficiencies combined with untrainability regardless of a registration as medical specialist abroad.	Yes
2016-63259 H	No longer having the support of the nursing staff on the ICU after discarding promises of restricting patient contact leading to safety risks.	Yes

Legend: This table shows the arguments used in 15 cases of residents whom were dismissed from residency because of compelling reasons. This concerned reasons other, or on top of illness or untrainability. Although concerns for patient safety were expressed in at least eight cases, we have no evidence that the patents in these cases actually suffered from the reported incidents, because the training staff discovered these incidences in time. The conciliation board respected compelling reasons to dismiss in twelve of the fifteen cases, but refused some arguments about patient safety as a reason, were as the board respected other reasons to dismiss. C = Community-based Practice, H = Hospital-based Specialty

## Discussion

After summarizing our main findings in the section below, we subsequently hypothesise about the findings, and discuss how these findings contrast and complement the literature on residents’ remediation and dismissal.

### Main findings

This ten-year nationwide case law study of dismissed residents demonstrated a different chance of success in appeals by residents from community-based practice versus hospital-based specialties. The most alleged reason to dismiss a resident, in general, was lack of trainability, with risks for patient safety mentioned less frequently. In community-based practice, residents less successfully appealed their dismissal at the Dutch conciliation board. The remediation strategies differed between the two groups. Community-based specialties used more flexible forms of remediation, with written notes about the specific requirements for residency continuation, in contrast to hospital-based specialties, which used more formal remediation plans with strict timing, and suffered from more omissions of technicalities.

### Discussion of main findings

*Comparison.* Appeals of residents from community-based practice appeared significantly less likely to succeed when compared to hospital-based specialties. The resident groups did however not differ regarding residents’ characteristics such as years of training or number of deficient CanMEDs competencies. Whereas residents’ characteristics were comparable in both groups, differences nevertheless exist between programme policy characteristics. Programme policy characteristics present in community-based practice, but not in hospital-based specialties, weekly mentor/reflectional classes, the possibility for a resident to disconnect the supervising relationship prematurely, and request a new supervisor, quarterly progress meetings, and conditional passing [10]. The regularly scheduled reflection classes provide input and documentation about the residents’ patterns of professional behaviour. In addition, the head of residency training is mandated and allowed to dismiss the resident from training with flexibility in timing, unlike hospital-based specialties where dismissal due to untrainability is only allowed during formally scheduled summative assessment meetings. So, community-based practice has different, and more ductile educational policies regarding assessment, remediation, and dismissal compared to hospital-based specialties. We therefore hypothesize, in the apparent absence of residents’ individual factors explaining the findings, that policy characteristics of community-based training practice might contribute to a higher rate of dismissal confirmation by the conciliation board, even in case of discard of formalities or technicalities.

*Remediation strategies.* The remediation strategies described in the current study are representative of the classic reflecting practices described by Steinert & Levitt in 1993 [35] regarding the remediation of medical expertise, communication, and management. In the cases of the current study, little attention was paid to root cause analysis (RCA, such as described by Arnold et al., 2016) [25] of competency deficiencies. RCA implies a structured non-judgemental analysis of roots and causes performed in case of a notification of a pattern of deficient performance from a resident. This analysis includes investigating the context of the reporters and the educational climate itself, followed by systematically optimizing support tailored to the needs of residents and training staff. Such an analysis might have been valuable to the residents in our law cases, but was not reportedly used in any of them. Remediating medical expertise in the current study mostly concerned increasing exposure to tasks and skills residents were inexperienced with and specific assessment of these skills in practice. An attempt was made to improve the resident’s communication skills with video supervision, role play, communication training, and assessment; for residents of foreign descent, there was a specific dedication to improving the Dutch language in speech (comprehension, expression, pronunciation) and writing (grammar, spelling, vocabulary). Remediating deficiencies in management skills were mostly about timely or gradual reduction of work and educational demands. Surprisingly, teaching time management skills by course or example were never mentioned. Furthermore, communication was mostly seen as an isolated CanMEDs competency instead of a competency connected to other competencies. The programme directors showed little aspiration to improve the residents’ generic skills such as social skills training in these case law descriptions. It is however possible that residents’ needs were nevertheless not completely acknowledged or met in remediation. We know from the literature that deficiencies in interpersonal skills, such as a shortage of social skills, might predict remediation needs [36], so it seems logical to target these deficiencies in remediation plans. However, professionalism deficiencies, such as problematic interaction, introspection, involvement, or integrity [37] might be especially hard to remediate [6, 33]. In the case law of the current study, first the resident received an oral motivational about observed deficiencies which were transcribed, a practice similar to Hickson et al. (2006) [38]. The most common way in which professionalism deficiencies in the law cases was dealt with, was to write or talk about them (with a mentor, coach, tutor, or therapist) to promote reflection. Specific reflection-promoting therapies such as mentalization-based [39] and mindfulness [40] were never mentioned in the law cases. New remediation strategies have been published meanwhile [41], for example applying simulation-based professionalism training [42].

*Reasons to dismiss.* Lack of trainability was the most alleged reason to dismiss a resident. Lack of trainability is defined as a failed remediation attempt as concluded by the programme directors because of the lack of learning progress of the resident during training. Dismissed residents in community-based practice had persisting deficits in professionalism, whereas residents dismissed from hospital-based specialties were more often untrainable in aspects associated with the medical expertise domain. Lack of trainability could result from residents’ characteristics, the residency training programmes’ quality, and/or resident - residency programme mismatch. Persistent sick leave was accepted as a reason for dismissal in the case law. Sick leave could cause and/or contribute to the residents’ untrainability due to inability to participate in training as well as in remediation. Sick leave was unaccustomedly frequently reported in the case law in both community-based and hospital-based specialties. In some cases, sick leave could be a consequence of pressure to perform during a remediation attempt or a dismissal procedure. In other cases, sickness had been persistent or frequent, obstructing the trainability of the resident. Sickness causing intermittent absence in shifts might hinder the residents’ reliability or collegiality. Residents may struggle handling their own illness within the work ethics in medicine, where the norm presses residents to be present and strive for excellence.

Residents lacking reliability from the perspective of the training staff were prevented to participate in shifts, emergency, or ICU wards through immediate dismissal for compelling reasons. As evidenced by the case law descriptions, the training staff appeared not to tolerate a combination of unreliability, untrainability, and incompetence in unpredictable environments with high demand for acute and critical emergency interventions [11]. Perceived future risks for patient safety were indeed accompanied reasons for dismissal in our case law, however, no incident was reported causing actual damage to patients. Fortunately, the staff was attentive enough to prevent causing harm.

*Reasonable judgment*. When do dismissed residents deserve a second chance? A reasonable judgement about residents’ dismissal may depend on the severity and/or frequency of identified issues displayed despite feedback. Some single incidents may be sufficient grounds for dismissal, such as sexual harassment of patients, drug use when on call, or theft– the type of conduct that seriously interferes with the resident’s credibility regardless of the origin of the behaviour. Likewise, other issues which appeared of minor importance, may have significant impact due to the high frequency of occurrence, such as condescending behaviour towards patients or colleagues, declining of help in a crisis situation, inability of theory of mind, or failure to report a transfer, which all may compromise the quality of patient care. Reasonableness must at least include considerations of institutional culture, and the relevant factors revealed in the current study such as: personal problems or illness, risks to patient safety, duration of the training, and the match with the remediation programme including accurate communication and documentation about deficiency severity and resident guidance.

#### Strengths

To the best of our knowledge, we herein present the results of a unique nationwide study of case law in the context of postgraduate medical education. The characteristics of dismissed residents have seldom been studied in such detail, and even more limited in comparison with different specialties [43]. In addition, information about mediation and conciliation attempts and strategies in residency training is confidential in most countries, and studies on this topic so far lacked completely. Our study included a large number of residents (*n* = 70) for such a type of study and combined use of a case-control design correcting for differences in educational policy with judicial open source data, which is a novum in postgraduate medical education research.

#### Limitations

This study has however several limitations. Information about dismissed residents who did not appeal is unfortunately unavailable in the Netherlands. So we had no possibility to compare our data of those who appealed with that of all the dismissed residents. The resident cases were anonymized by the conciliation board themselves, so the investigators were not able to identify the residents, and had to limit their research questions to the data available. Furthermore, the number of law cases per specialty as the basis for statistical analysis was consequently small.

#### Recommendations and ideas for further research

We recommend the education governing bodies and the programme directors to optimize educational policies of hospital-based specialties including remediation methods, using the regulations and strategies applied in community-based practice as an example. While being “untrainable” is an umbrella explanatory term for dismissal in Dutch postgraduate medical education, this untrainability seems to be more specifically associated with persisting deficiencies in professionalism in community-based practice and deficiencies in medical expertise in hospital-based specialties. Studies exploring the generalisability of our findings by replication of this study in other countries are needed.

## Conclusion

The most commonly alleged reason to dismiss a resident was a lack of trainability followed by endangerment of patient care. Residents from hospital-based specialties were reportedly more likely to succeed after a dismissal appeal, when compared to residents from community-based practice. This contrast in procedural success may result from differences in characteristics in educational policy. Compared to hospital based specialties, community-based practice appears to have a more structured and well documented assessment programme of the residents’ performance including quarterly progress meetings, and precise documentation about the specificity, and severity of residents’ deficiencies. Moreover, community-based practice offers flexible remediation strategies to residents, such as conditional passing, disconnection of resident and supervisor, weekly reflectional classes that provide insight and documentation about the resident professional development, and ultimately, the educational regulation in community-based practice allows to dismiss an untrainable resident at any moment of their residency. The remediation and dismissal policies in hospital-based specialties may be improved by mirroring procedures in place in community-based practice.

### Electronic supplementary material

Below is the link to the electronic supplementary material.

Supplementary Material 1

Supplementary Material 2

## Data Availability

All data was anonymized by The Royal Dutch Conciliation Board before published publicly available online: https://www.knmg.nl/opleiding-herregistratie-carriere/rgs/wat-doet-de-rgs/bezwaar-beroep-en-geschil/geschillencommissie-geschillenprocedure/uitspraken-en-jaarverslagen-geschillencommissie.htm#Jaarverslagen_Geschillencommis_(Uitspraken_en_jaarverslagen_Ge)-anchor. (Retrieved 23 January 2022). Coded data is available from the corresponding author on reasonable request.
