# Supplementary material for: Dutch dismissal practices: characteristics, consequences, and contrasts in residents’ case law in community-based practice versus hospital-based specialties
Source: BMC Med Educ. 2024 Feb 19;24:160. doi: 10.1186/s12909-024-05106-w (PMC10877891; doi:10.1186/s12909-024-05106-w)
Supplement: Supplementary file 1 — Supplementary Material 1 [file 12909_2024_5106_MOESM1_ESM.docx]

Appendix Consort Diagram

2011-2020

229 requests for conciliation

98 were ultimately withdrawn by the applicants, resulting in unpublished decisions

3 inadmissible, because filed too late

8 cases not about dismissal

50 cases excluded, previously in training for psychiatry, occupational medicine, intellectual disability physician, neurosurgery, otolaryngology, gynecology, orthopedics, nuclear medicine, pathology, microbiology, rehabilitation medicine, neurology, emergency medicine.

70 cases

previously in training for: community-based practice.

previously in training for hospital-based specialties.
