# Supplementary material for: Dutch dismissal practices: characteristics, consequences, and contrasts in residents’ case law in community-based practice versus hospital-based specialties
Source: BMC Med Educ. 2024 Feb 19;24:160. doi: 10.1186/s12909-024-05106-w (PMC10877891; doi:10.1186/s12909-024-05106-w)
Supplement: Supplementary file 2 — Supplementary Material 2 [file 12909_2024_5106_MOESM2_ESM.docx]

**Selection and matching of case law of hospital-based specialties.**

The hospital-based cases were matched by systematic and randomized exclusion of available law cases.

First we decided not to include cases in psychiatry and rehabilitation medicine, because those specialties are not strictly hospital based in the Netherlands.

Second we decided not to include cases in pathology and medical microbiology because those specialities do not treat their own hospital patients. They treat patients only on a consultation basis, via other hospital specialties.

Third we wanted a mix of cases from specialties in surgery, internal medicine and diagnostic/supportive specialties. Table 2 provides the proportion of law cases in relation to the total number of residents in training. We decided to pick larger specialties with at least four law cases.

The residue of law cases not included were: neurosurgery, neurology and otolaryngology (all just one law case), nuclear medicine and gynecology (each three cases or less), orthopedics, (four cases not selected being a subspecialty).

See below the list of cases included of hospital-based specialities:

RGS-GC 10 oktober 2011, 11-63158, anesthesiology

RGS-GC 13 augustus 2012, 12-63178, anesthesiology

RGS-GC 18 november 2013, 13-63204, anesthesiology

RGS-GC 13 november 2014, 14-63231, anesthesiology

RGS-GC 12 juli 2016, 16-63255, anesthesiology

RGS-GC 23 augustus 2016, 16-63259, anesthesiology

RGS-GC 4 november 2019, 19-08, anesthesiology

RGS-GC 8 november 2012, 12-63187, dermatology

RGS-GC 10 december 2012, 12-63189, dermatology

RGS-GC 7 november 2016, 16-63262, dermatology

RGS-GC 13 juni 2017, 17-63275, dermatology

RGS-GC 10 juli 2017, 17-63277, dermatology

RGS-GC 1 september 2015, 15-63241, surgery

RGS-GC 23 januari 2018, 18-05, surgery

RGS-GC 1 oktober 2019, 19-07, surgery

RGS-GC 10 april 2017, 17-63271, surgery

RGS-GC 26 september 2017, 17-63281, internal medicine

RGS-GC 17 mei 2016, 16-63252, internal medicine

RGS-GC 5 januari 2012, 11-63164, internal medicine

RGS-GC 14 februari 2017, 17-63268, internal medicine

RGS-GC 17 mei 2011, 11-63144, radiology

RGS-GC 28 juni 2011, 11-63148, radiology

RGS-GC 11 juli 2012, 12-63177, radiology

RGS-GC 24 maart 2014, 14-63213, radiology

RGS-GC 10 juli 2014 & 8 januari 2015, 14-63233 & 14-63221, radiology

RGS-GC 30 september 2014, 14-63225, radiology

RGS-GC 8 oktober 2016, 16-63263, radiology

RGS-GC 17 juli 2017, 17-63279, radiology

RGS-GC 30 november 2017, 17-63286 radiology

RGS-GC 21 juni 2018, 18-95, radiology

RGS-GC 24 juni 2019, 2019-4 radiology

RGS-GC 22 december 2011, 11-63163, radiotherapy

RGS-GC 20 maart 2014, 14-63211, radiotherapy

RGS-GC 20 december 2016, 16-63266 radiotherapy

RGS-GC 4 oktober 2018, 18-143, radiotherapy
